# Nanoscale chirality in metal and semiconductor nanoparticles

**DOI:** 10.1039/c6cc05613j

**Published:** 2016-08-17

**Authors:** Jatish Kumar, K. George Thomas, Luis M. Liz-Marzán

**Affiliations:** a CIC biomaGUNE , Paseo de Miramón 182 , 20009 Donostia – San Sebastián , Spain . Email: jatishkumar@gmail.com ; Email: llizmarzan@cicbiomagune.es; b School of Chemistry , Indian Institute of Science Education and Research Thiruvananthapuram (IISER-TVM) , CET Campus , Thiruvananthapuram , 695 016 , India; c Ikerbasque , Basque Foundation for Science , 48013 Bilbao , Spain

## Abstract

The field of chirality has recently seen a rejuvenation due to the observation of chirality in inorganic nanomaterials. The advancements in understanding the origin of nanoscale chirality and the potential applications of chiroptical nanomaterials in the areas of optics, catalysis and biosensing, among others, have opened up new avenues toward new concepts and design of novel materials. In this article, we review the concept of nanoscale chirality in metal nanoclusters and semiconductor quantum dots, then focus on recent experimental and theoretical advances in chiral metal nanoparticles and plasmonic chirality. Selected examples of potential applications and an outlook on the research on chiral nanomaterials are additionally provided.

## Abstract 2

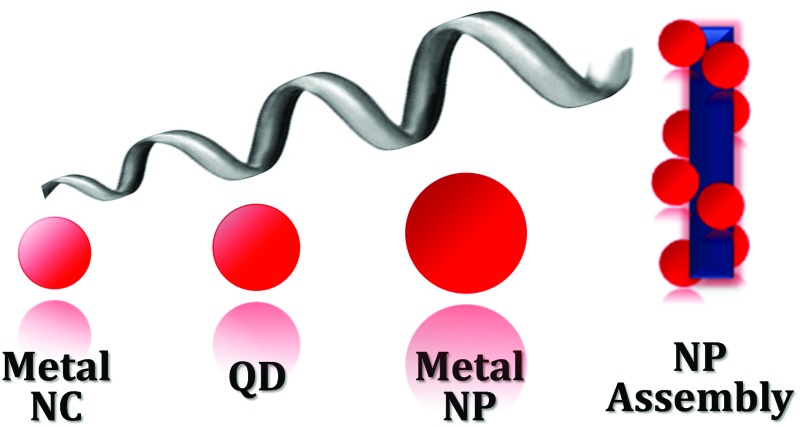
We discuss optical activity in metal nanoclusters and semiconductor quantum dots, broadly focusing on recent advances in nanoscale chirality in plasmonic nanoparticles and their assemblies.

## Introduction

The vast variety of chiral objects in the universe, ranging from galaxies^1^ to sea shells^2^ to human DNA^3^ and amino acids,^4^ has fascinated researchers for decades. Indeed the mere fact that the chiral objects in nature have a preference for one handedness over another has always been captivating for mankind in general and scientists in particular.^5,6^ Fascinated by the symmetry principles of natural objects, architects have created chiral structures with well defined handedness (for example, Chicago Spire, Mode Gakuen spiral tower, La Défense tower and Gherkin building).^7^ Our interest in understanding the physical, chemical, and biological properties of chiral objects traces back to the observation of optical activity in quartz in 1811.^8^ The art of chiral design was first translated from macroscopic structures to molecular building blocks in the laboratory bench by Louis Pasteur, way back in 1848, through his seminal work on left- and right-handed crystals of sodium ammonium tartrate that could bend polarized light in clockwise and anticlockwise directions.^9^ Since then, a plethora of activities have been observed towards unraveling the structures of chiral biomolecules including proteins, amino acids and DNA, design of newer chiral molecular systems and the investigation of their physical properties.^3,4,10,11^ Mankind has also experienced the devastating consequences of neglecting the biological effects of chirality while using thalidomide as a drug in the early 1960s.^12^

Any structure can be defined as chiral if it is asymmetric in such a way that the structure is non-superimposable with its mirror image. Optical activity in molecules or materials arises due to the differential interaction of the medium to left and right circularly polarized light. This differential interaction can be in the form of electronic absorption, vibrational absorption, reflection or fluorescence. Based on the phenomena involved, various techniques such as electronic circular dichroism (ECD),^13^ vibrational circular dichroism (VCD),^14^ diffuse reflection circular dichroism (DRCD)^15,16^ and circularly polarized luminescence (CPL)^17,18^ have been developed. While the CD based techniques provide information on the ground state chirality of the molecules, CPL being related to luminescence gives information on the excited state chirality. These techniques together can function as a powerful tool to provide stereochemical, conformational and three-dimensional structural information of chiral molecules and materials in both the ground and excited states. The chiral dissymmetry in any object will largely depend on the efficiency of the sample to differentiate between left and right circularly polarized light. The optical activity in any system is quantified using the anisotropy factor or the *g* factor which is given by the equation *g* = Δ*ε*/*ε*, where Δ*ε* and *ε* are the molar CD and molar extinction, respectively.

**Fig. 1:**
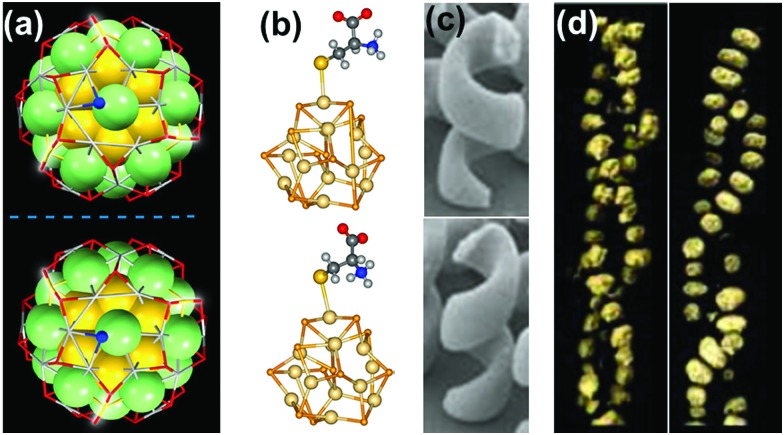
Chirality in metallic, semiconductor and plasmonic nanostructures and assemblies: (a) X-ray structure of enantiomers of [Ag_46_Au_24_(SR)_32_](BPh_4_)_2_ NCs. Reproduced with permission from ref. 58. Copyright 2015 American Association for the Advancement of Science. (b) Optimized geometries of l- and d-cysteine capped semiconductor CdSe nanocrystals. Reproduced with permission from ref. 74. Copyright 2013 American Chemical Society. (c) TEM images of left- and right-handed gold nanohelices. Reproduced with permission from ref. 89. Copyright 2009 American Association for the Advancement of Science. (d) TEM tomography images of left- and right-handed gold NP double helices formed on a peptide template. Reproduced with permission from ref. 132. Copyright 2013 American Chemical Society.

The majority of chiral systems synthesized and probed in the laboratory till 1998 were based on organic molecules.^10,18^ These studies include design of organic and bio-molecular systems showing macroscopic chirality.^18–20^ Novel approaches in the design of inorganic systems showing chiroptical properties at the nanoscale have provided rejuvenation to this branch of science.^21^ Various inorganic nanostructures such as TiO_2_,^15^ CuO,^16^ and ZnO^22^ were introduced which exhibit optical activity based on reflection, refraction or electronic transition. However, efforts in the direction of creating inorganic chiral nanoobjects are largely directed towards combining the optical properties of chiral molecules with metal or semiconductor nanoparticles (NPs).^23–26^ This involves the synthesis of metal or semiconductor NPs that are stabilized with chiral organic or bio-molecules as stabilizing ligands. Researchers in this field have tried to focus on the origin of chirality at the nanoscale by investigating (i) the effect of chiral ligands on altering the core structure of NPs leading to the formation of a chiral core; and (ii) the interaction of the ligand and NP electronic states leading to the creation of new hybridized states and thereby new optical properties. Interestingly, such nanoobjects with chiroptical properties are proposed as potential candidates for a variety of applications. These include (i) the design of nanoscale materials having negative refractive indexes (metamaterials)^27^ and (ii) chiral sensing, catalysis and synthesis.^28,29^ In this feature article, we briefly discuss optical activity in metal nanoclusters (NCs) and semiconductor NPs, and broadly focus on the recent advances in the nanoscale chirality of plasmonic NPs and their assemblies (Fig. 1).

## Chiral metal nanoclusters

Metal NCs constitute an interesting class of materials that were used at the early stages of the study on chiral NPs.^30–32^ These are few metal atom clusters possessing attractive properties such as small size, high molar absorptivity and photostability. Most studies on metal clusters are currently focused on gold and silver NCs.^33–35^ Metal NCs are generally synthesized by reduction of metal salt precursors with mild reducing agents in the presence of a stabilizing ligand. Metal NCs exhibit well-defined absorption peaks and characteristic CD bands in the visible range when stabilized using chiral ligands. A classic study that initiated the investigations on chirality at the nanoscale was that by Schaaff and Whetten on Au_28_ clusters stabilized using glutathione as the chiral ligand.^36^ The formation of lower symmetry chiral structures that would minimize the total energy of Au clusters was shown to be the basis of the observed optical activity.^37^ Various groups have subsequently reported experimental and theoretical findings on chiral NCs that shed light on the evolution of chirality in these nanoscale materials.^38–40^

Chirality in metal NCs can be generated from either an intrinsically chiral core or an achiral core in a chiral environment. The chiral environment in the latter case can be created by chiral ligands, or by an asymmetric arrangement of achiral ligands. Based on these mechanisms, different models have been developed to explain the source of chirality in NCs:^30^ (i) the chiral core model, in which chirality is generated by an intrinsically chiral core, or arises from structural distortion due to the interaction with the chiral ligand;^37,41–43^ (ii) the dissymmetric field model, which is characterized by an achiral core with chirality induced either through a chiral adsorption pattern or through vicinal effects by chiral ligands;^34,44–48^ (iii) the chiral footprint model, in which a local chiral distortion of the surface atoms is achieved through the adsorption of the ligand.^49–51^ Based on the models discussed above it is clear that both chiral and achiral ligands can induce chirality in metal NCs.^51,52^ These models are formulated on the basis of the extent to which chiral dissymmetry of the ligand is transmitted to the atomic structure of metal NPs. Although the proposed models have been supported by experimental data, the detailed mechanism of the origin of chirality in metal clusters is not sufficiently understood.

**Fig. 2:**
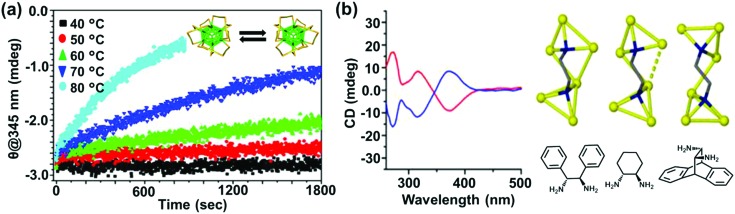
(a) CD response at 345 nm for the racemization of enantiopure Au_38_(SCH_2_CH_2_Ph)_24_ at different temperatures. The response is negative at 345 nm and approaches zero with time; the rate increases with temperature. The inset shows the schematic representation (created using crystallographic data) of the racemization reaction along the principal axis of the cluster. Reproduced with permission from ref. 53. Copyright 2012 American Chemical Society. (b) CD spectra and model of enantiopure metal cluster complexes. Molecular structures of the three 1,2-diamine ligands (one enantiomer) used for the synthesis of chiral clusters. Reproduced with permission from ref. 55. Copyright 2016 American Chemical Society.

The most straightforward approach toward generating chirality in NCs comprises the use of chiral ligands. In addition to breaking the symmetry of the surface, chiral molecules influence the surface atoms to create a local handedness that can strengthen the asymmetry of the NC. Bürgi and co-workers used a variety of thiols and showed that the adsorbed chiral ligands can affect the asymmetric arrangement of surface gold atoms in Au NCs.^38^ Inversion of surface chirality was demonstrated through ligand exchange using the opposite enantiomer of the thiolated ligand.^50^ The authors also investigated the racemization of an intrinsically chiral Au_38_ nanocluster which showed drastic rearrangement of the cluster surface involving place exchange of several thiolates. Racemization takes place at modest temperatures (40–80 °C), and the activation energy for the inversion reaction was calculated to be 28 kcal mol^–1^ indicating that the process occurs without complete breaking of the Au–S bond (Fig. 2a).^53^ Tsukuda and coworkers used Au_11_ clusters to demonstrate the role of chiral phosphine ligands in altering the arrangement of surface metal atoms or the entire metal core, thereby significantly influencing the chiroptical properties.^54^ In a recent report, Zhao and coworkers used a series of chiral metal clusters stabilized with chiral amines to get better insights into the role of the chiral ligands in determining the spatial arrangement of metal atoms, and further tried to probe the correlation between the metal atom arrangement and the chiroptical property (Fig. 2b).^55^

**Fig. 3:**
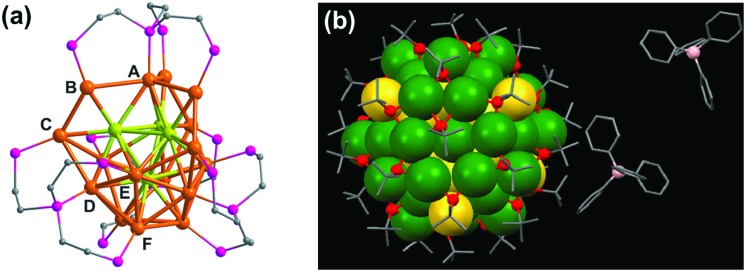
(a) X-ray crystallography structure of the chiral gold NC [Au_20_(PP_3_)_4_]Cl_4_ (PP_3_ = tris(2-(diphenylphosphino)ethyl)phosphine), showing the bridging mode of PP_3_. The 16 surface gold atoms are of six types (from A to F), the four interstitial Au atoms are shown in green, and phenyl groups are omitted for clarity. Reproduced with permission from ref. 41. Copyright 2014 Wiley-VCH. (b) X-ray crystallography structure of one of the enantiomers of a bimetallic chiral [Ag_46_Au_24_(SR)_32_](BPh4)_2_ NC. Gray: carbon; red: sulfur; green: silver; yellow: gold; pink: boron. The hydrogen atoms are omitted for the sake of clarity. Reproduced with permission from ref. 58. Copyright 2015 American Association for the Advancement of Science.

A second approach that has been adopted to synthesize chiral clusters is based on using achiral ligands. In a recent report on phosphine ligand induced chirality, an intrinsically chiral Au_20_ core was generated using an achiral polydentate phosphine ligand tris(2-(diphenylphosphino)-ethyl)phosphine (Fig. 3a).^41^ In a similar work on Au NCs, the X-ray structure of Au_102_(*p*-MBA)_44_ (*p*-MBA = *para*-mercaptobenzoic acid) showed a chiral pattern for the arrangement of the gold–sulfur staple motifs on the cluster surface despite the use of an achiral ligand.^52^ Interestingly, the asymmetric pattern of thiolate ligands around an achiral core could generate strong Cotton effects in CD.^56^ Optical activity was also recently observed in Au_38_(SCH_2_CH_2_Ph)_24_ gold clusters, wherein chirality is generated from the chiral arrangement of achiral thiolates.^57^ The enantiomers could be successfully separated by chiral high-performance liquid chromatography (HPLC) and characterized by mirror-image CD responses with large anisotropy factors. Apart from single metal clusters discussed above, another class of materials that is recently gaining interest is related to core–shell structures comprising different metals. Large Ag–Au alloy NCs consisting of an achiral bimetallic Ag_2_@Au_18_@Ag_20_ core protected by a chiral Ag_24_Au_6_(SR)_32_ shell have been reported by Wang and coworkers (Fig. 3b) and characterized using X-ray crystallography.^58^ Garzón and co-workers carried out theoretical calculations toward the geometric quantification of the origin of chirality in intrinsic chiral clusters. They found that an asymmetric arrangement of surface metal atoms plays a major role in the chirality of the whole cluster.^59^

## Chirality in semiconductor nanocrystals and their assemblies

**Fig. 4:**
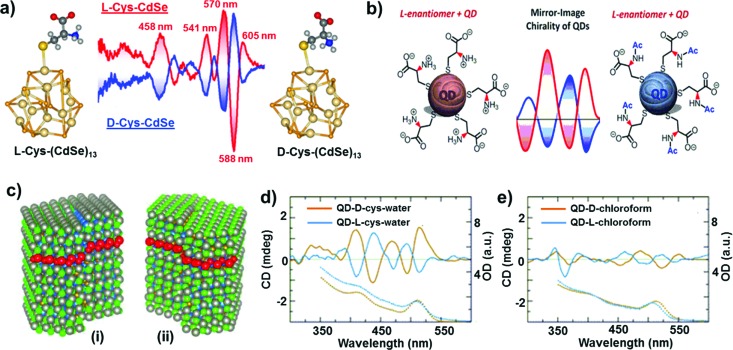
(a) Induced CD spectra of l- and d-cysteine capped CdSe QDs. Optimized geometries of the corresponding nanocrystals are also provided. Adapted with permission from ref. 74. Copyright 2011 American Chemical Society. (b) Schematic representation of l-cysteine (left) and *N*-acetyl-l-cysteine (right) capped CdSe QDs along with the corresponding CD spectra obtained for the two NPs showing signal inversion. Adapted with permission from ref. 75. Copyright 2016 American Chemical Society. (c) Atomistic models for CdSe/ZnS QDs with right (i) and left (ii) screw dislocations. (d and e) Spectra of induced (d) and intrinsic (e) CD in l- and d-CdSe/ZnS QDs. Absorption spectra are shown as dotted lines. Adapted with permission from ref. 60. Copyright 2015 American Chemical Society.

In comparison to chiral metal NCs, the investigations on chiral semiconductor NPs or quantum dots (QDs) are relatively new, and they have not been widely explored. Chirality in semiconductor QDs can be explained based on the three mechanisms described for chiral induction in metal NCs:^25,26^ (i) NPs with a chiral core, (ii) NPs with an achiral core and chiral surface due to chiral distortion or ligands adsorbing in a chiral pattern, (iii) NPs with both achiral core and achiral surface where the chiroptical effect arises due to electronic interactions between the capping ligands and the inorganic core. Due to the crystallization of semiconductor NPs into achiral space groups, the mechanism involving a chiral core was ruled out in this class of materials until the recent finding of chirality in CdSe/ZnS QDs due to defects in the chiral core.^60^ Early studies on the optical activity of semiconductor NPs involved synthesis in the presence of chiral stabilizing ligands. Optical activity was first demonstrated in CdS QDs stabilized with penicillamine as the capping ligand.^61^ The NPs maintained an undistorted and achiral core, whereas CD signals arise from near-surface cadmium atoms that are enantiomerically distorted by chiral ligands. Various other chiral semiconductor NPs were synthesized using chiral and achiral capping ligands that exhibited characteristic CD responses at and outside the band-edge region of the spectrum.^62–72^ An interesting report claimed that CdTe QDs exhibited chiral memory effects, meaning that the chiral surface could be retained even after ligand exchange of chiral l- and d-cysteine methyl ester with achiral 1-dodecanethiol.^73^ Recently, induced CD and CPL have been observed upon simple phase transfer of achiral trioctylphosphine oxide (or oleic acid) capped CdSe QDs, from toluene into an aqueous phase containing chiral l- or d-cysteines (Fig. 4a).^74^ The QDs exhibited size-dependent CD that originated from the hybridization of chiral ligand molecular orbitals with QD valence band states. Inversion of the QD CD signal was also demonstrated by altering the structure of the ligands without changing their absolute configuration (Fig. 4b).^75^ Apart from such ligand induced chirality, intrinsic chirality in CdSe/ZnS QDs and quantum rods (QRs) stabilized by achiral ligands was recently reported by Mukhina *et al.*, which was ascribed to naturally occurring chiral defects. However, intrinsic CD for both QDs and QRs was found to be much weaker than induced CD signal intensities (Fig. 4c–e).^60^

**Fig. 5:**
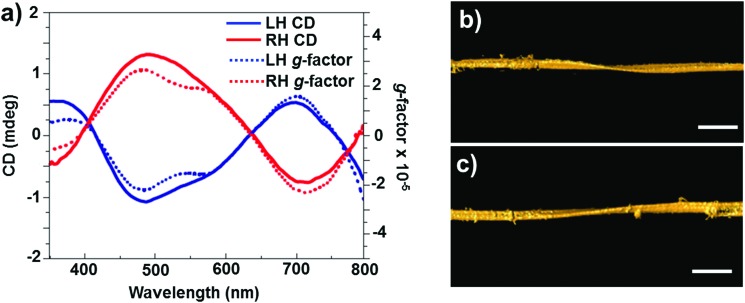
(a) CD (solid line) and *g*-factor (dotted line) spectra for solutions of left- and right-handed nanoribbons obtained after 50 h of illumination with circularly polarized light. (b and c) Representative 3D TEM tomographic reconstructions of left- (b) and right-handed (c) nanoribbons. Scale bars = 100 nm. Adapted with permission from ref. 76. Copyright 2015 Nature Publishing Group.

In addition to chirality observed from individual particles, semiconductor NP assemblies also exhibited enhanced optical activity. Recently, Kotov and coworkers demonstrated that circular polarization of light can strongly influence the assembly of QDs by altering the chirality of particles involved in a self-assembly process.^76^ Illumination of a racemic solution of CdTe NPs with right- and left-handed circularly polarized light induced the formation of right- and left-handed twisted nanoribbons, with an enantiomeric excess above 30% (Fig. 5), while straight nanoribbons formed upon exposure to linearly polarized light or in the dark. Illumination of the racemic solution (containing equal amounts of left- and right-handed particles) with light of a specific polarization gave rise to enantioselective photoactivation of specific NPs and clusters (*e.g.* when illuminated by left circularly polarized light, a subpopulation of left handed NPs and clusters absorb light more efficiently than right handed ones and *vice versa*). The authors also showed that activated NPs undergo ligand photooxidation, resulting in bare CdTe particles that can self-assemble into nanoribbons with specific helicity, as a result of chirality-sensitive interactions between the NPs. The same group recently demonstrated that chiral CdTe NPs coated with cysteine self-organize into helical supraparticles. d- and l-Cysteine determines the dominant left- and right-helicity of the supraparticles, which further self-organize into lamellar crystals with a liquid crystalline order.^77^ Recent theoretical investigations on semiconductor nanowires have demonstrated that optical activity can be inherent to such nanowires, similar to the chiral induction by dislocations developing during the growth.^78^

## Chiral plasmonic nanomaterials

Among the nanostructured materials that exhibit chirality at the nanoscale, plasmonic NPs are rapidly emerging as a potential front-runner due to their multidisciplinary applications in the fields of chemistry, biology, medicine and materials science.^79,80^ The peculiar size and shape dependent properties of localized surface plasmon resonances in plasmonic nanomaterials can help tuning chiral signatures over the whole visible and near-IR range.^23–26^ Owing to the small size of NCs or QDs, surrounding chiral ligands may induce structural changes in the core atoms resulting in chiral cores. In contrast, chiral ligands can only influence the arrangement of surface atoms in the case of NPs with larger size. Moreover, it is difficult to alter the crystalline nature of the internal core for producing chiral structures. Hence, the investigation on chiral noble metal NPs with larger sizes (ranging from 5 to 100 nm) is more challenging as compared to that on small metal clusters or QDs. Investigations on the optical activity of plasmonic nanomaterials can potentially unravel the underlying structural information of these materials. Based on the origin of optical activity arising from either individual particles or NP assemblies, chirality in plasmonic nanomaterials can be divided into three classes.^79,80^ Chirality in individual particles can be due to either (i) the inherent chiral shape of the particles or (ii) the interaction of chiral molecules with achiral NPs. In contrast, nano-assemblies can exhibit chirality due to (iii) the interaction of achiral NPs in a chiral configuration. Moreover, a large fraction of plasmonic NPs under investigation are non-spherical particles, which in turn contribute to additional asymmetries in NPs and assemblies, and hence to chirality.

## Chiral shaped particles

**Fig. 6:**
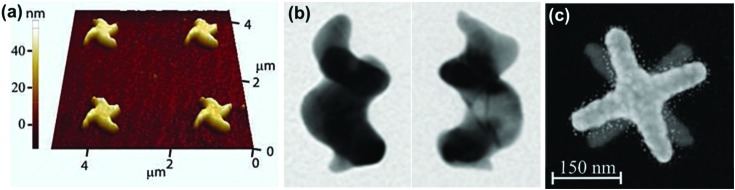
(a) Atomic force microscopy image of right-handed star-shaped gold nanostructures that were made chiral by curved, wedge-shaped spiral arms. Reproduced with permission from ref. 92. Copyright 2012, Optical Society of America. (b) TEM images of left- and right-handed gold nanohelices possessing two turns. Adapted with permission from ref. 93. Copyright 2013 Nature Publishing Group. (c) Electron micrograph of left-handed twisted-cross gold nanodimers consisting of two achiral crosses on top of each other. Reproduced with permission from ref. 94. Copyright 2011, Optical Society of America.

Optical activity is to be expected from plasmonic nanomaterials of sub-wavelength dimensions possessing chiral morphology. The optical response would be in favor of the dominant enantiomer and its magnitude will depend on the extent of electronic delocalization. The basic concepts of chirality that were initially developed for organic molecules are currently being transferred to metal nanostructures. Hence, optical activity in chiral shaped nanostructures can be related to the spatial chirality exhibited by derivatives of helicenes, coronenes and biphenyls.^81,82^ As compared to organic systems, plasmonic nanomaterials have the advantage of possible electron delocalization over a larger surface, and hence can exhibit more intense optical response. Nanoengineering strategies have been developed for fabricating plasmonic materials with chiral shapes, which mainly involve top-down approaches such as electron beam lithography,^83,84^ glancing angle deposition,^85,86^ hole-mask colloidal lithography^87^ and direct laser writing.^88,89^ Optical activity in individual nanostructures with chiral shapes could be generated by fabricating propeller and spiral shaped structures, exhibiting helical chirality.^88–93^ For example, helical gold nanostructures possessing four curved arms were fabricated using electron beam lithography (Fig. 6a).^92^ The star-shaped structures exhibited strong nonlinear CD. Spiral gold nanostructures in 3D were also synthesized mimicking helical spiral-shaped molecules (Fig. 6b).^93^ Mirror image CD spectra with distinct positive and negative first Cotton effects were recorded for right- and left-handed nanohelices, respectively. In another example, individual twisted-cross gold nanodimers fabricated through layer by layer electron beam lithography showed strong chiroptical behavior, both at the fundamental wavelength and at the second harmonic (Fig. 6c).^94^ In the sample, each of the gold crosses was achiral but chirality arises from the mutual orientation of the crosses in the dimer, indicating that chirality is associated in this case with the 3D character of the nanostructures. The above mentioned techniques allowed a wide range of applications for these nanostructures in the fields of metamaterials,^95,96^ circular polarizers^89^ and biomolecular sensing.^97^

**Fig. 7:**
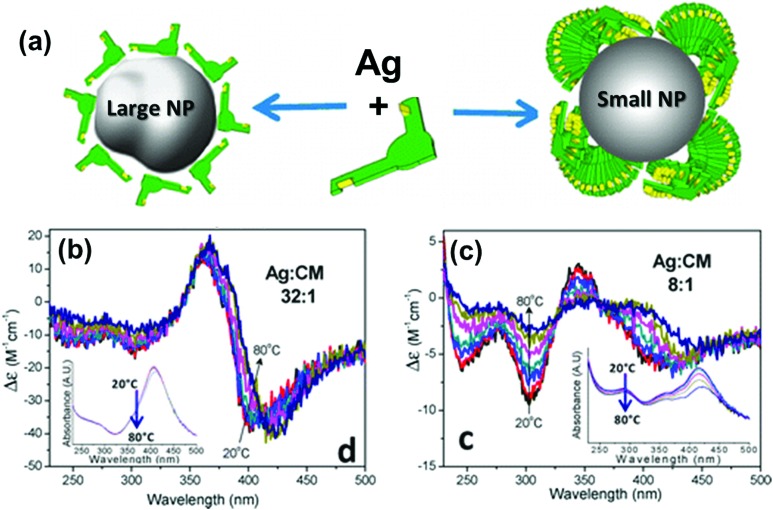
(a) Schematic illustration of a large Ag NP covered by a monolayer of molecules and a small NP stabilized with large molecular stacks. (b and c) CD spectra of Ag NPs coated with chiral molecules prepared from Ag : molecule ratios of (b) 32 : 1 (large NP) and (c) 8 : 1 (small NP), measured as a function of temperature. Insets show the respective extinction spectra, which undergo only small changes with temperature. Adapted with permission from ref. 99. Copyright 2012 American Chemical Society.

Chirality arising from the shape of NPs has also been observed in colloidal nanostructures. Shape effects can give rise to CD in the corresponding plasmonic region, which can even arise from small deformations of the NPs. Hence, bisignate CD signals at the plasmon resonance wavelengths, along with TEM tomography,^98^ can be used as effective methods to study the chiral deformation in NPs. The insensitive nature of plasmonic CD towards temperature changes has been used to confirm the formation of chiral shaped Ag NPs stabilized using self-assembled structures of chiral molecules (Fig. 7).^99^ At higher temperature, where the molecular assemblies dissociate, small Ag NPs showed a decrease in plasmonic CD whereas larger particles maintained their plasmonic CD intensity. This indicates that the observed chiroptical effect in larger particles is not induced by the molecular monolayer. Model calculations suggest that the plasmonic CD may be the result of a slight chiral shape distortion and creation of structurally chiral silver NPs.

## Interaction of chiral molecules with achiral NPs

**Fig. 8:**
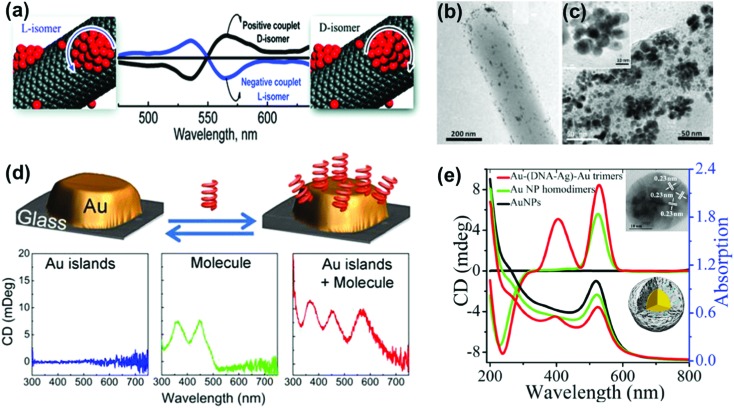
(a) Bisignated CD signals observed at the surface plasmon frequency with positive and negative couplets for NP bunches adsorbed on the surface of d- and l-diphenylalanine nanotubes. (b and c) TEM images of diphenylalanine peptide nanotubes (b) in the presence of Au NP seeds and (c) after photochemical irradiation for 3 h in the presence of HAuCl_4_ for reductive growth of NPs on the seeds. The inset shows the HRTEM image of one of the NP bunches. Adapted with permission from ref. 101. Copyright 2010 American Chemical Society. (d) Schematic illustration of plasmonic CD induction by chiral riboflavin molecules in the vicinity of gold islands deposited on a glass substrate. Chiral molecules induce optical activity in Au islands leading to CD signals at the metal plasmon resonance of 570 nm. Adapted with permission from ref. 107. Copyright 2013 American Chemical Society. (e) CD and UV-vis spectra of Au–(DNA–Ag)–Au trimers, and control experiments. The inset shows a TEM image and a scheme of the core–shell trimer nanostructure. Reproduced with permission from ref. 109. Copyright 2015 Wiley VCH.

**Fig. 9:**
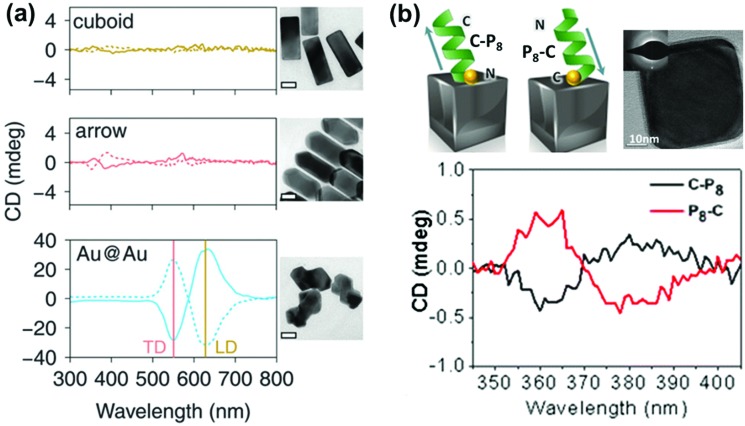
(a) CD spectra of Au@Ag nanocuboids and nanoarrows with cysteine adsorbed on their surfaces and Au@Au NPs grown from cysteine modified Au nanorods (cysteine at the core–shell interface). Solid and dotted lines represent l- and d-cysteine, respectively. TEM images of the different Au@Ag NPs are also shown (scale bar = 20 nm). Reproduced with permission from ref. 110. Copyright 2016 Royal Society of Chemistry. (b) Schematic illustration of the inverse adsorption of a peptide on the surface of a nanocube, along with a high resolution TEM image of the cube. CD spectra of the nanocube with C-P_8_ (cysteine unit at the N terminus – black trace) and P_8_-C (cysteine unit at the C terminus – red trace). Reproduced with permission from ref. 111. Copyright 2016 American Chemical Society.

Optical activity can be induced at the electronic states of achiral NPs when they interact strongly with a chiral molecule. A clear observation of plasmonic CD was first reported by Kotlyar and coworkers in silver NPs grown on chiral double stranded DNA.^100^ Bisignated CD signals were later observed at the surface plasmon frequency with positive and negative couplets for NP bunches adsorbed on d- and l- diphenylalanine nanotubes, respectively. The organization of Au NPs in two different ways was driven by the chirality of the constituent molecules in the nanotubes (Fig. 8a–c).^101^ A large number of studies on hybrid systems involving noble metal NPs and chiral molecules have been reported thereafter.^102–105^ A wide variety of chiral biomolecules (DNA, amino acids, proteins and peptides) along with chiral organic molecules were used to induce CD at LSPR wavelengths.^100–111^ In an interesting report, the effect of the metal–chiral molecule distance on the intensity of the induced CD was studied using Au islands (Fig. 8d).^107^ The induction was found to decay within about 10 nm from the surface for 33 nm Au islands. Recent studies on metallic core–shell structures showed that enhanced optical responses can be generated by entrapping chiral molecules at the core–shell junctions.^108–110^ Chiroptical activity was observed in Au core-DNA–Ag shell NPs wherein the structures exhibited CD activity at the LSPR region with a high *g*-factor (Fig. 8e).^109^ The CD response of the individual NPs could be controlled by altering the Ag shell thickness. Unusual chiroptical effects were also observed at hot-spots produced by creation of inter-particle junctions. Enhancement of plasmonic CD has been recently reported in cysteine capped Au NRs *via* Ag coating.^110^ The entrapment of cysteine at the Au–Ag interface amplifies the local electromagnetic field and leads to strong enhancement of the CD response (Fig. 9a). An additional advantage from anisotropic NPs was emphasized by the fact that no plasmonic CD signals were observed in spherical core–shell NPs. Plasmonic CD from silver nanocubes could be inverted by altering the orientation of chiral molecules (peptide) with respect to the surface of the nanocube (Fig. 9b).^111^ The effect was correlated to multipolar effects at plasmon resonances involving hot spots created at nanocube apexes and edges.

Detailed theoretical analysis on the various mechanisms involved in the induction of plasmonic CD has been carried out by Govorov and coworkers.^112–115^ Metal–chiral molecule hybrid systems can function in a complementary fashion; achiral plasmonic NPs can enhance the CD responses of chiral molecules whereas chiral molecules can induce new optical responses at the LSPR wavelengths of NPs. While the former effect can be amplified by using nanostructures with a strong plasmonic enhancement, such as NP pairs and nanoshells, the latter effect helps in shifting the molecular chirality from the UV into the visible spectral range. Metal–chiral molecule interactions have been investigated by formulating different models based on the strength of interaction and the size of the particles. For small NP systems, the near-field plasmonic CD induction model based on a quasi-static approximation is adopted. The amplitude of the plasmon CD peak rapidly decreases with interparticle separation and varies depending on the orientation of the molecular dipole with respect to the NP surface. This model has been applied to single spherical NPs, NP pairs and nanoshells.^112,113^ For larger NPs with sizes around half the wavelength (>100 nm), the plasmon CD induction is formulated on the basis of the far field electromagnetic coupling mechanism. The CD response in this case is induced by the interaction of the plasmonic mode with the chiral parameters of the medium surrounding the NP. This method is utilized to calculate the CD induction in systems such as achiral plasmonic particles with a chiral shell, chiral plasmonic particles with an achiral shell and various anisotropic systems.^114,115^

## Achiral NPs interacting in a chiral configuration (plasmonic NP assemblies)

**Fig. 10:**
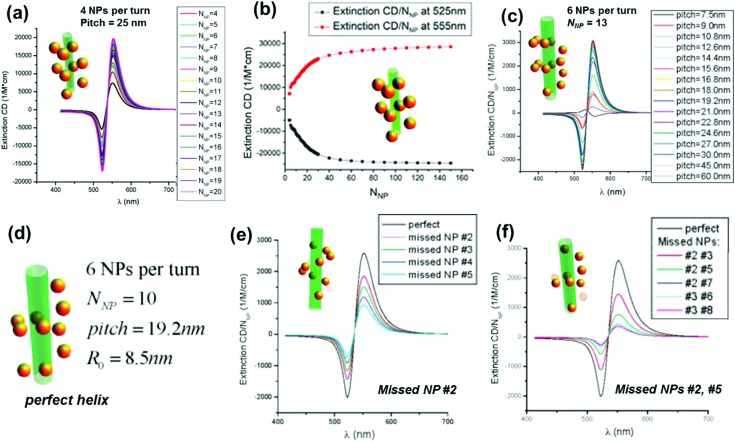
(a) Calculated normalized extinction CD, *ε*_extin,CD_/*N*_NP_, for optimized Au-NP helices with pitch = 25 nm. Other parameters: NP radius (*a*_NP_) = 5 nm and helix radius (*R*_0_) = 12 nm. (b) Calculated normalized extinction CD at two selected wavelengths *λ* = 525 and *λ* = 555 nm. The selected wavelengths correspond to the extreme points of the CD spectra. (c) Calculated normalized extinction CD for 6-NP-per-turn helices with a variable pitch and a constant helical radius *R*_0_ = 8.5 nm; number of NPs (*N*_NP_) = 13; and *a*_NP_ = 2.5 nm. (d–f) Calculated normalized extinction CD for 6-NP-per-turn helices with various defects: a model of a perfect helix (d), the helix with one missing NP (e), and the helices with two missing NPs (f). The parameters are pitch = 19.2 nm, *N*_NP_ = 10, *a*_NP_ = 2.5 nm, and *R*_0_ = 8.5 nm. Adapted with permission from ref. 119. Copyright 2011 American Chemical Society.

One of the widely used methods to generate chirality in achiral nanomaterials is through the arrangement of particles in a chiral fashion leading to plasmon coupling. The strength of induced optical activity is largely dependent on the size of the particles as well as the aggregates. Interaction of molecular dipoles leading to exciton coupling and bisignate CD is well studied and documented.^116^ Mimicking the exciton coupling in organic chromophores, theoretical investigations were carried out on dimers of plasmonic nanoantennas.^117^ In contrast to molecular systems where the extent of interaction is limited, the rather large scale dipolar and multipolar interactions possible in plasmonic nanomaterials render them better candidates for chirality generation through this approach. Numerical calculations of the plasmonic CD response of interacting achiral nanoparticles were carried out using classical dipole–dipole electromagnetic theory.^118,119^ Considerable plasmonic CD was observed from systems possessing some extent of helical correlations between the NP positions. The calculations indicated that the CD responses were sensitive to the composition as well as the geometry of the NP assemblies, and the intensity decreases with the occurrence of defects (Fig. 10).^119^ Optimized parameters for designing novel assemblies with strong optical chirality that are stable against defects or disorder were calculated by modeling the influence of helix parameters on the CD signal.

**Fig. 11:**
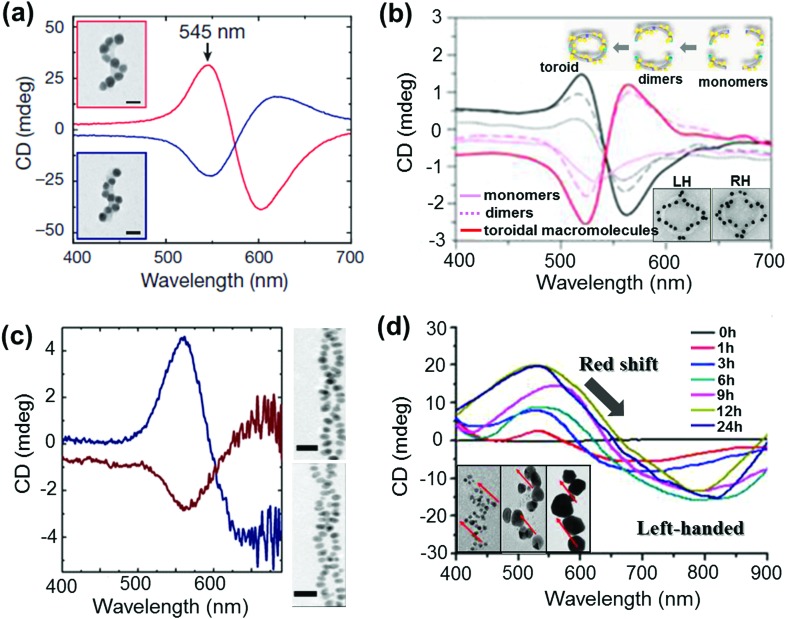
(a and b) DNA origami based templates: (a) CD spectra of left- (red lines) and right-handed (blue lines) helices made of nine gold NPs with 16 nm diameter, showing characteristic bisignate signatures in the visible region (the position and intensity of the peaks could be drastically changed by varying the size of the particles). Adapted with permission from ref. 123. Copyright 2012 Nature Publishing Group. (b) CD spectra of plasmonic right-handed (RH – red traces) and left-handed (LH – black traces) helical monomers, dimers, and toroidal metamolecules. Scheme of the stepwise formation of plasmonic toroidal metamolecules and TEM images of LH and RH toroids (inset). Adapted with permission from ref. 125. Copyright 2016 American Chemical Society. (c) Peptide template; CD spectra and TEM tomography image of left-handed (blue trace, top image) and right-handed (red trace, bottom image) gold NP double helices. Adapted with permission from ref. 132. Copyright 2013 American Chemical Society. (d) Organic self-assembled templates; CD spectra of a hydrogel containing one equivalent of molecules and three mol equivalents of Au(i) ions after increasing durations of UV irradiation for reductive growth of gold NPs on the helical nanofibers. Inset: TEM images after UV irradiation for 1 h, 6 h and 24 h show the increase in particle size with time. Adapted with permission from ref. 145. Copyright 2014 American Chemical Society.

NP assemblies that are capable of generating coupled plasmon CD are generally fabricated on organic or biomolecular templates wherein the functionalities on the template play a crucial role in positioning the NPs and thereby controlling the coupling interactions. The technique demands the formation of relatively large assemblies that possess strong inter-particle interactions and well defined handedness. This makes the selection of suitable templates and particles rather more challenging. Initial works on chiral assemblies focused on simple helical and pyramidal structures constructed from nanoparticle assemblies. While no optical activity was observed from the initial structures,^120,121^ CD responses were later observed from structures containing four NPs of different sizes, in analogy to tetrahedral carbon.^122^ The CD intensity in the case of assembled structures was much stronger than that reported for many individual NPs with adsorbed chiral molecules. The solution based approach makes use of various soft templates such as DNA,^122–130^ peptides,^131,132^ lipids,^133^ cellulose,^134,135^ amino acids,^136,137^ chiral liquid crystals,^138–140^ silica^141–143^ and various organic self-assembled structures.^144–146^ Kuzyk *et al.* observed large optical activity in chiral NP assemblies adsorbed on DNA origami scaffolds. The CD signals arise from the helical assembly of strongly interacting gold NPs (Fig. 11a).^123^ DNA origami scaffolds have been widely used for the synthesis of NP assemblies with different structure and geometry. Helical monomer, dimer and toroidal structures comprising DNA origami and gold NPs were synthesized and shown to exhibit plasmonic CD (Fig. 11b).^125^ Several other hybrid systems made of metal NPs and chiral soft templates were designed and synthesized, exhibiting plasmonic CD which was generated from the chiral arrangement of metal NPs. Some recent examples include formation of double helical NP assemblies on peptides (Fig. 11c)^132^ and UV induced reduction of gold salt on self-assembled molecular fibers to form helical NP assemblies (Fig. 11d).^145^ In addition to the widely used solution based self-assembly approaches, some of the techniques used to fabricate helical NP assemblies include electron-beam lithography, direct laser writing and glancing angle deposition.^147,148^

**Fig. 12:**
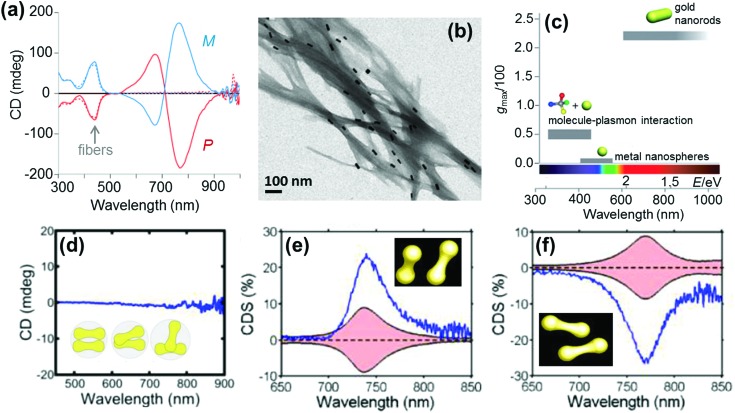
(a) CD spectra of P (red) and M (blue) nanocomposites. (b) TEM image of the M nanocomposite exhibiting twisted fibers with nanorods adsorbed on the surface. (c) Representative values of anisotropy factors and the corresponding typical spectral ranges for metal NPs in fluid media. Reproduced with permission from ref. 146. Copyright 2011 Wiley VCH. (d) Ensemble CD spectra of the Au nanodumbbell dimer (no peaks were observed). The inset shows the cartoon depiction of the dimers. (e and f) Experimental single particle CD scattering spectra of the two Au nanodumbbell dimers with twisted side-by-side geometry (the dimers show opposite signs, as expected for two enantiomers). Adapted with permission from ref. 149. Copyright 2016 American Chemical Society.

**Fig. 13:**
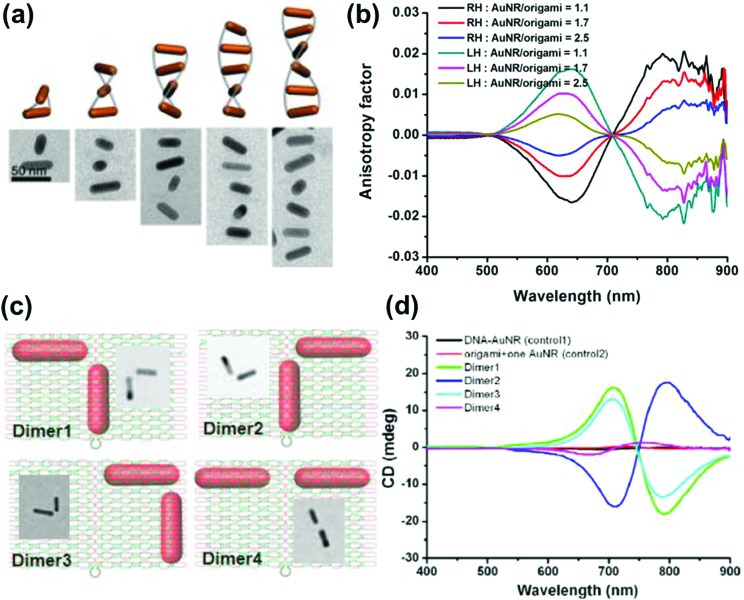
(a) Schematic illustration and cryo-TEM images of assembled right-handed AuNR helices on DNA origami with varying number of rods. (b) Measured CD anisotropy factors of right-handed (RH) and left-handed (LH) AuNR helices corresponding to the structures in the image. Reproduced with permission from ref. 127. Copyright 2015 American Chemical Society. (c) Schematic illustration and TEM images of AuNR dimers on quasi-2D DNA origami. (d) CD spectra corresponding to the dimers in (c), along with their controls. Reproduced with permission from ref. 128. Copyright 2016 American Chemical Society.

Anisotropic nanorods (NRs) are interesting candidates for plasmon coupled CD induction, since they allow for organization of individual components in different configurations, and facilitate the tuning of plasmonic CD signals by simply varying the aspect ratio of helically arranged NRs.^127–130,135–137^ Moreover, theoretical calculations have also suggested significantly enhanced optical activity for prolate ellipsoidal particles in the helix relative to their spherical counterparts.^146^ In one of the earlier attempts on using nanorods for generation of surface plasmon CD, a large bisignated Cotton effect was observed at the plasmon coupled frequency for Au NRs adsorbed on supramolecular fibres possessing chiral morphology (Fig. 12a–c).^146^ The results were interpreted on the basis of the coupled-dipole model. In a different approach, assemblies of Au NRs in an end-to-end and side-by-side fashion were constructed by using cysteine as the linker. Interestingly, side-by-side assembled NRs exhibited stronger plasmonic CD response compared with the end-to-end assemblies, which was attributed to the change in electromagnetic interactions between NRs in the assemblies.^136^ Recently, tailored chirality was demonstrated from helical superstructures of Au NRs assembled on a 2D DNA origami template. Right- and left-handed helices were synthesized, and the number of rods in the helices could be tailored by varying the origami : rod molar ratio. This helped in the delicate tuning of the resulting chiral signal intensity (Fig. 13a and b).^127^ More recently, strong plasmonic chirality was observed from quasi-2D gold NR dimers assembled on soft DNA origami and the sign as well as the intensity of the chiral response was dependent on the configuration of the dimers (Fig. 13c and d).^128^ The plasmonic chirality was far more sensitive to the spatial variations of the dimers when compared to their plasmon absorption. In an interesting work by Link and coworkers, gold nanodumbbells having plasmonic properties similar to those of their NR counterparts were used to generate plasmonic CD response. Whereas the dispersion of dimers prepared using achiral components showed no detectable ensemble CD response, single-particle circular differential scattering (CDS) measurements revealed that the dimer sample is a racemic mixture of individual nanostructures with significant positive and negative chiroptical signals (Fig. 12d–f).^149^

## Applications of chiral plasmons

**Fig. 14:**
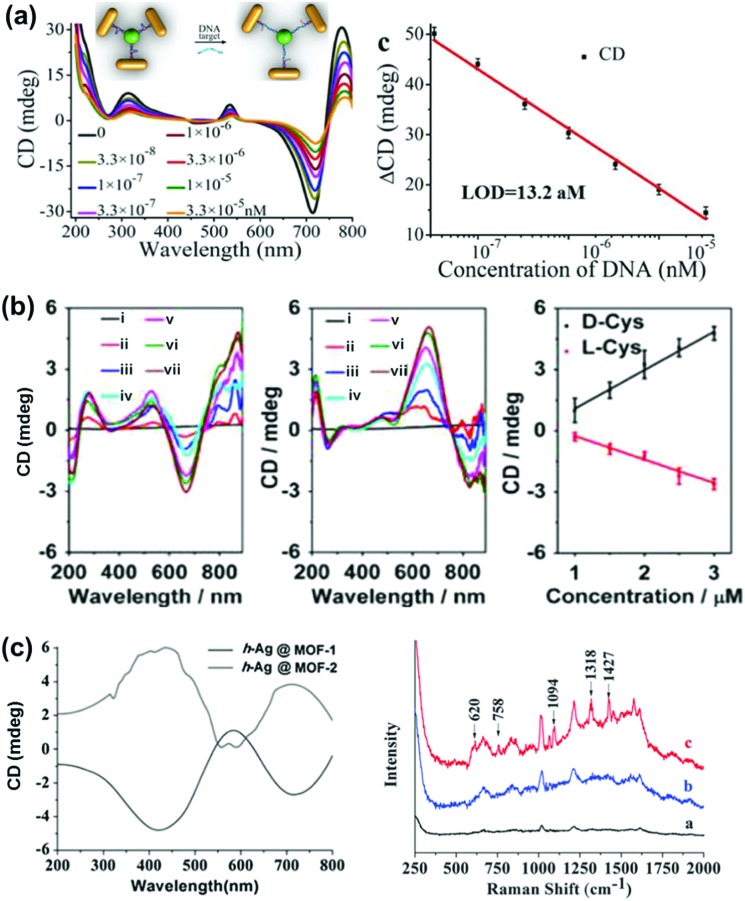
(a) DNA detection using NR tetramer assemblies. CD curves with increasing concentrations of DNA solution and the CD calibration curves for DNA detection. The longitudinal absorption peak of the NRs used for assembly was 750 nm. The inset shows the schematic illustration for DNA biosensing. Adapted with permission from ref. 151. Copyright 2016 Wiley VCH. (b) Cysteine and glutathione sensing using NRs. CD spectral changes of Au NRs upon addition of l- and d-cysteine. The concentration of cysteine is (i) 0, (ii) 1, (iii) 1.5, (iv) 2.0, (v) 2.5, (vi) 3.0 and (vii) 5 μM. Linear relationship between CD signals at 670 nm and the added concentration of d-Cys or l-Cys. Adapted with permission from ref. 154. Copyright 2016 American Chemical Society. (c) SERS sensor based on Ag NPs@homochiral MOF. CD and the corresponding SERS spectra of (i) h-Ag NPs@MOF-1, (ii) h-Ag NPs@MOF-2 with 1.0 μmol L^–1^ d-cysteine and (iii) h-Ag NPs@MOF-1 with 1.0 μmol L^–1^ l-cysteine. Reproduced with permission from ref. 157. Copyright 2016 Royal Society of Chemistry.

NP–chiral molecule hybrid systems and chiral nano-assemblies with optical activity in the plasmonic region have attracted great interest in the field of biosensors, nanodevices, surface-enhanced Raman scattering (SERS) and other photonic applications.^28,29,150–157^ After directing considerable attention to the synthesis and mechanistic details of chiral NPs, researchers are currently focusing their attention on finding potential applications for these chiral nanomaterials. In a recent report by Wu *et al.*, propeller shaped nanoscale tetramers exhibiting strong chiroplasmonic and enhanced upconversion luminescent properties were fabricated, which could be utilized for DNA detection with unusually low level of detection (Fig. 14a).^151^ In a similar work, a different approach involving a combination of plasmonic CD and fluorescence from DNA–gold pyramid hybrids was used for ultrasensitive and selective intracellular detection of microRNA in real time.^152^ The plasmonic CD signals were much more sensitive to the concentration of miRNA than the luminescent signals, indicating that chiral nano-assemblies can function as potential probes for intracellular bioanalysis. Liu *et al.* developed a simple and sensitive CD based method for the detection of methyltransferase activity and inhibition by using DNA-driven self-assembled AuNP dimers as probes.^153^ The method could be successfully applied in complex matrices such as human serum samples with high accuracy and precision. End-to-end assemblies of gold nanorods with chiral cysteine or glutathione were used as a chiroptical sensor for reliable determination of the absolute configuration of the biomolecules in the visible light region. The sign and the amplitude of the Cotton effect were explored to correlate with the absolute configuration and concentration of the chiral biomolecules (Fig. 14b).^154^ In a similar approach, chiral plasmons arising from the side by side assembly of gold NRs were successfully utilized for the attomolar detection of DNA.^28^ Recently, Tang and coworkers reported that Ag NPs arranged in a helical structure on a chiral metal–organic framework can serve as a new type of SERS sensor for the efficient recognition of d/l-cysteine and d/l-asparagine enantiomers (Fig. 14c).^157^ In addition to the above mentioned applications, chiral shaped materials fabricated by the top down approach can function as chiral metamaterials. Due to their appealing properties, such as negative refraction and photonic band gaps, metamaterials have potential applications as super-lenses, invisibility cloaks, broad-band circular polarizers, *etc.* ^27,89,95–97^

## Summary and outlook

In summary, we have discussed the recent advances in nanoscale chirality focusing on metal and semiconductor NPs. We briefly discussed the recent approaches adopted for the synthesis and chiroptical studies of chiral metal NCs and semiconductor nanocrystals. The mechanisms developed for chirality evolution in metal NCs were based on an (i) inherent chiral core and (ii) achiral core surrounded by a chiral environment. Similar effects in semiconductor QDs give rise to CD responses in the band edge region, and this could in most cases be explained on the basis of the distortion of surface atoms by chiral ligands or through defects in the nanocrystals. The wide focus of the article is on the chirality in plasmonic nanomaterials, which can be broadly divided into three classes: (i) chirality arising from the inherent morphology of individual particles, (ii) chirality induced on achiral NPs through chiral ligands attached on the surface, and (iii) chirality in achiral particles arising from the favorable interparticle coupling interactions. Each of the three cases has been supported by adequate experimental reports and further complemented with advanced theoretical calculations. The final part focuses on the potential applications of chiral plasmons.

While we have focused our attention on plasmonic, metal and semiconductor particles, it is obvious from the above discussion that nanoscale chirality can be exhibited by a wide variety of systems ranging from organic to inorganic particles and assemblies. The strength of chiral signals is not controlled solely by properties inherent to the material, but depends largely on the arrangement of constituent elements within the particle. One of the challenging tasks with materials at the nanoscale is the production of nanostructures that exhibit consistent and reproducible properties. This can only be achieved through a clear understanding of the mechanisms involved. Therefore, research on unraveling the underlying mechanisms of chirality in nanomaterials is currently a hot research topic. The mere fact that chirality can be induced in structures by breaking mirror or inversion symmetries may prompt one to assume that formulating the mechanism is a straightforward task. However, it must be noted that there is no single mechanism that can describe the origin of chirality in nanostructures. A variety of different mechanisms may be at work in each of the nanostructures, which may function complementarily or independently. Moreover, the existence of nanostructures possessing a wide variety of sizes and shapes, along with their assembled structures, makes it possible to break the symmetry in a large number of ways. Theoretical calculations are extensively used these days in conjunction with the experimental results, so as to understand the mechanism of chirality in nanomaterials. Moreover, potential applications that these chiral nanomaterials can find in the fields of optics, biology and chemistry have attracted much interest. Compared to chiral molecular systems, nanoscale chirality is a relatively new topic. Significant progress has been achieved during the last decade in the synthesis of nanomaterials possessing various morphologies and chiroptical properties. The major remaining challenge is the derivation of the mechanistic details and the use of these materials for practical applications. The availability of a wide variety of chiral systems allows researchers to go deeper into the origin of chirality and therefore potential applications will continue to attract the attention of nanomaterial scientists in the field of nanoscale chirality.
